# RoboticSurgery4all: are discovery courses important for robotic surgery skills acquisition?

**DOI:** 10.1007/s11701-024-02077-4

**Published:** 2024-08-14

**Authors:** Mário Rui Gonçalves, Björn Mück, Jean-Pierre Faure, Philippe Topart, Miguel Castelo-Branco Sousa

**Affiliations:** 1 Faculty of Health Sciences, Centro Académico Clínico das Beiras (Academic Clinical Center of Beiras), University of Beira Interior, Av. Infante D. Henrique, 6200-506 Covilhã, Portugal; 2 Klinikum Kempten, Klinikverbund Allgäu gGmbH, Kempten, Germany; 3 Department of Digestive Surgery, CHU Poitiers, University of Poitiers, Poitiers, France; 4 Clinique de l’Anjou, Angers, France

**Keywords:** Robotic surgery, Simulation, Education, Training, Bariatric surgery, Abdominal wall repair

## Abstract

Cost, logistics, and availability of robotic simulation opportunities suppose a real challenge for robotic surgery training. We aimed to test a new methodology for introduction to robotic surgery pre-congress courses. Two different “introduction to robotic surgery” pre-congress courses were developed. A new methodology using a sleeve/bypass, a ventral TAPP and an inguinal TAPP silicone models was implemented. After the session, the trainees answered a questionnaire to evaluate the course and the methodology using 1–5 Likert scales. A total of 21 participants participated in the courses and (72.2%) had no experience in robotic surgery. All trainees rated the course as good or excellent. There was a strong agreement between participants regarding the adequacy of the silicone models for this type of simulation/course. Trainees agree that the course gave them more confidence to perform a real robotic procedure, increased their interest in robotic surgery and made them feel ready to start their robotic surgery pathway. Congresses are a frequent way of contact between surgeons and robotic systems, mostly in the form of technical demonstrations or pre-congress courses. Our methodology showed that it is possible to allow for this contact in a low-cost way. This kind of courses is well received by congress delegates and have a positive educational impact. Despite of being "Discovery” courses, they have a positive impact on the congress, on the acquisition of robotic surgery skills and increase the interest in robotic surgery.

## Introduction

Robotic surgery (RS) is present in many operating rooms across the world, mostly the US and Europe with very promising results [1, 2]. There are many new robotic systems entering the market [3, 4] but, for now, only a few have enough data to demonstrate the real benefits of this technology on surgical outcomes and patient’s benefit [5]. Of course, experienced surgeons already are proficient in surgical procedures but the robot brings a new approach that has to be learned and mastered by the surgeon before using it on real patients, to assure the necessary safety and outcomes. Simulation plays an essential role on this and certification is also very important [6–8]. However, the cost, logistics and availability of robotic simulation opportunities suppose a real challenge to robotic learning and training [9]. Cadavers and animals are not an option when these activities are organized during congresses and technical demonstrations. We aimed to test a new course methodology using low-cost models for RS simulation.

## Methods

### Design

Two different pre-congress courses were developed (1 at IFSO 2024, Vienna, Austria, and 1 at EHS 2024, Prague, Czech Republic). Registration was supported by the robotic company and on a first-come-first-served basis. The goal of these *“Robotic Surgery Discovery courses”* was to introduce residents and surgeons to the technology; cover clinical aspects and surgical outcomes of robotic surgery; aspects and characteristics of the robotic system and the setup; and learn tips and tricks from the proctors of the courses. For 2024 courses, we kept the session distribution of previous courses but implemented a new methodology for the hands-on sessions, with virtual reality exercises and procedure-specific silicone models. The general program can be found in Table 1. After the sessions, the trainees answered a questionnaire to evaluate the models, the course and the methodology. The duration of the course was nearly 6 h, with nearly 1.5 h of lectures and 4.5 h of hands-on workshop and simulation.

**Table 1:** General program of the courses

Briefing	
Lecture 1	Training, set-up and steps to successful implementation
Lecture 2	Robotic-Assisted Surgery (RAS) outcomes
Hands-on session 1	- System overview - Port placement and docking - Instruments and accessories
Hands-on session 2	- Virtual exercises - Anatomic silicone models
Debriefing	

**Fig. 1:**
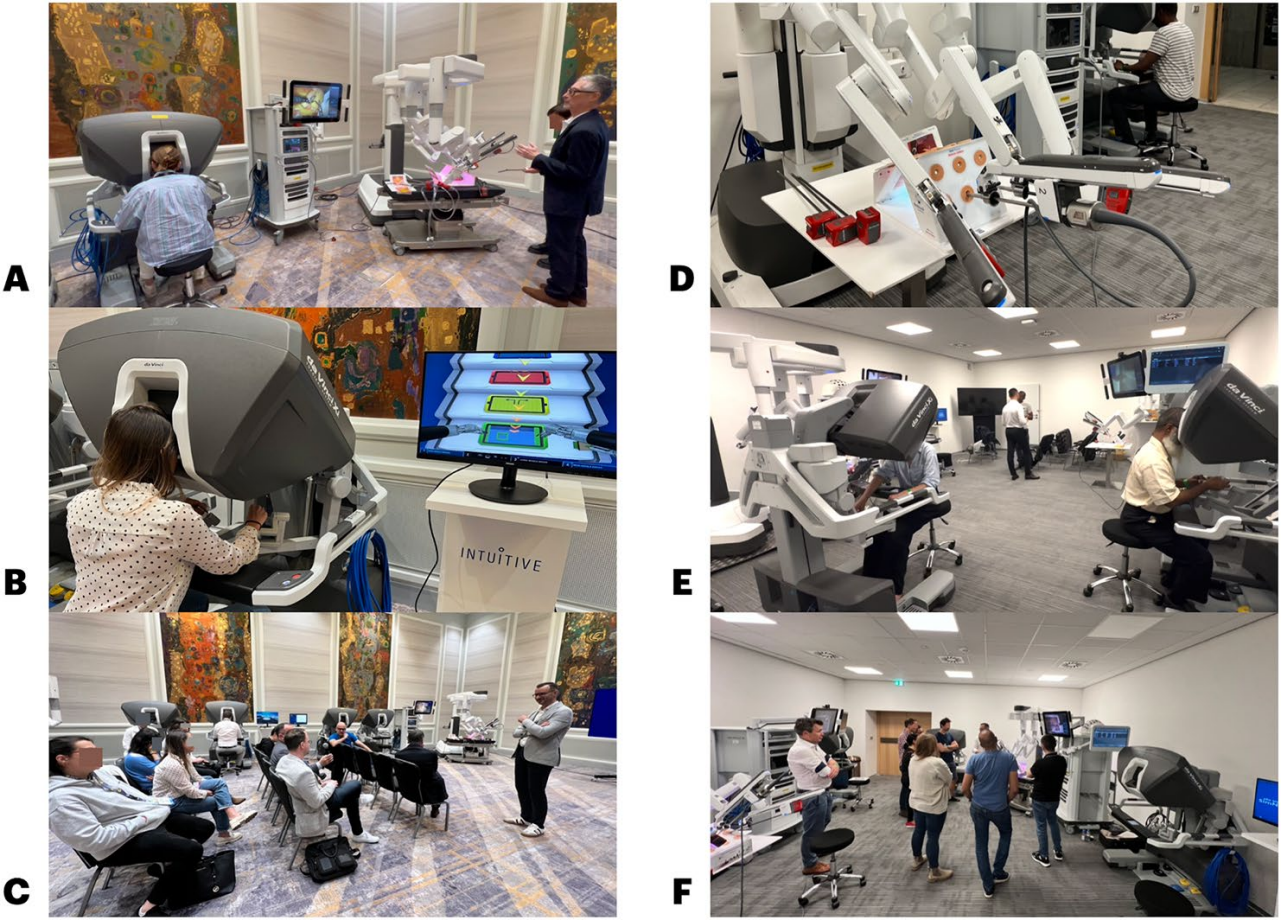
**A** View of a robotic system during a sleeve gastrectomy simulation (@IFSO 2024); **B** View of a robotic system during the VR exercises (@IFSO 2024); **C** View of the room during theoretical sessions (@IFSO 2024); **D** view of a robotic system during a Ventral TAPP repair simulation (@EHS 2024) **E** view of two robotic systems during the hands-on session (@EHS 2024); **F** view of the room during theoretical sessions (@EHS 2024)

Two robotic consoles were available during the sessions for the VR exercises’ session and two full robotic systems were used with the silicone models (Fig. 1A–F).

### Virtual reality exercises

Participants had to complete a sequence of, at least, five VR exercises: Sea Spikes, Camera Control, Wrist Articulation, Energy Pedals, Three Arm Relay.

### Simulation models

**Fig. 2:**
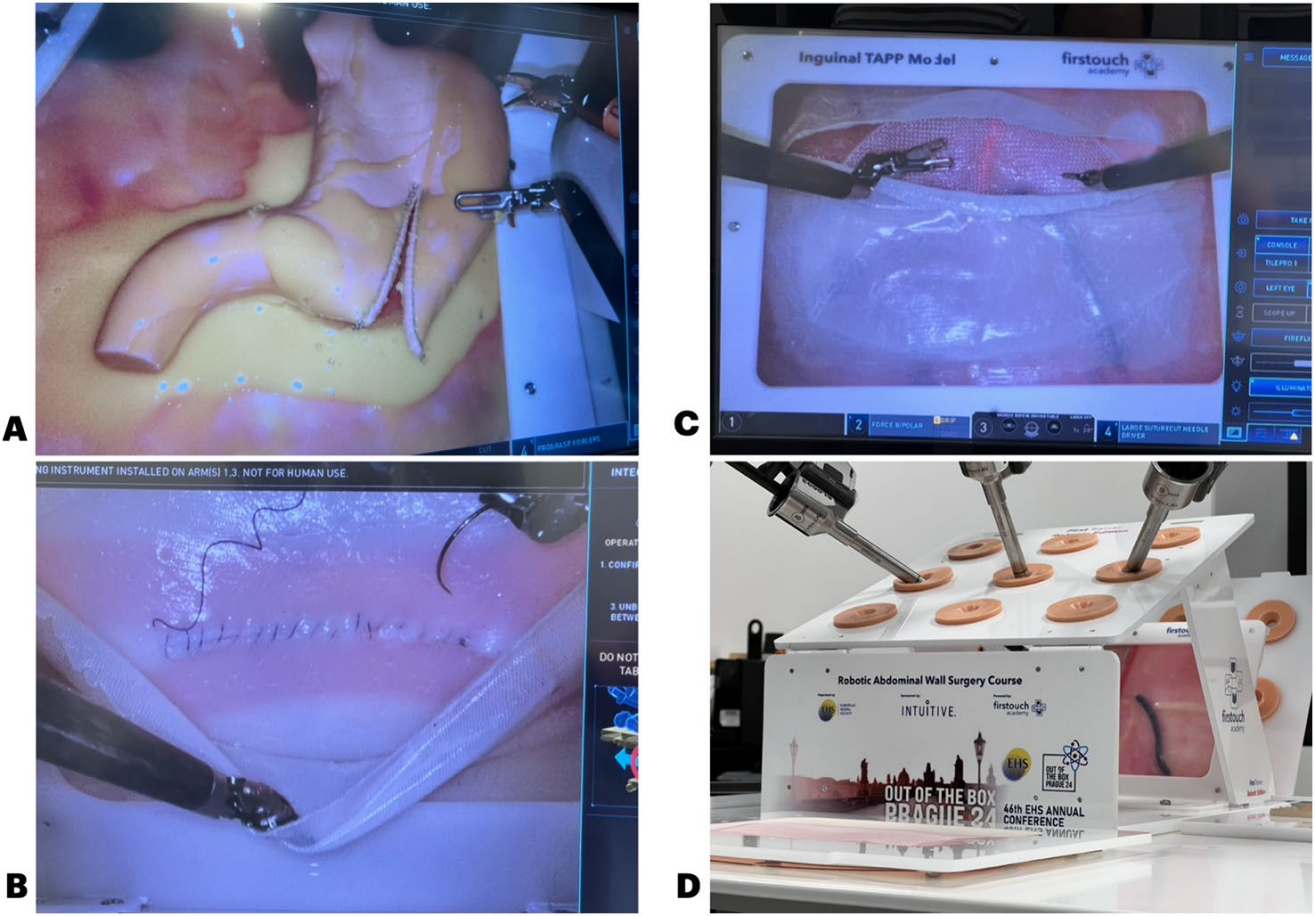
**A** Sleeve/Bypass model (@IFSO2024); **B** View of the ventral TAPP model (@EHS2024); **C** View of the inguinal TAPP model (@EHS2024); **D** View of the First Trainer, Robotic Edition (@EHS2024)

The silicone models used on these courses have been designed and produced by our team [10]. Insights from expert surgeons have been of extreme value to get all the features needed to simulate the procedure steps and the feeling of real tissues. Three different models were tested (Sleeve/Bypass model, Inguinal and Ventral TAPP repair model—Fig. 2A–D) and the robotic edition of the First Trainer® (Academia FT, Portugal) was used for the simulation on the robot (Fig. 2D) [11]. All the models were composed of multiple silicone layers, some of them with different texture and stiffness. This was the first time this version of the models have been tested on a real-world simulation, by surgeons outside our team.

### Questionnaires

Pre and post-course questionnaires have been developed and sent to the participants, using Google Forms® platform (Alphabet, INC, USA). Pre-course questionnaire contained open questions and statements regarding demographics, course expectations, previous RS experience, surgical background, among other information that can be found on Table 2. The Post-course questionnaire contained open questions and statements regarding the course, the methodology and the models evaluation, as well as other information that can be found on Tables 3, 4 and 5. The purpose of using open questions and statements was to obtain personal, authentic opinions of the participants, and avoid direct, biased questions about RS.

**Table 2:** Participants characteristics

	IFSO Course		EHS courses		Total	
	*n*	%	*n*	%	*n*	%
Total	8	45	10	55	18	100
Gender						
Male	3	37.5	3	30	6	33.3
Female	5	62.5	7	70	12	66.7
Age						
25–29	1	12.5	0	0	1	5.6
30–35	3	37.5	2	20	5	27.8
36–40	2	25	6	60	8	44.4
> 40	2	25	2	20	4	22.2
Main practice						
Public	8	100	8	80	16	88.9
Private	0	0	2	20	2	11.1
Grade						
Resident	2	25	2	20	4	22.2
Surgeon	6	75	8	80	14	77.8
Area of expertise						
Not Specific to the course	2	25	5	50	7	38.9
Specific to the course	6	75	5	50	11	61.1
Robotic surgery at hospital						
No	2	25	1	10	3	16.7
Yes, but not for the specialty of the course	5	62.5	7	70	12	66.7
Yes, for the specialty of the course	1	12.5	2	20	3	16.7
Robotic Surgery experience						
No experience	6	75	7	70	13	72.2
Discovery course	0	0	2	20	2	11.1
Intermediate course	1	12,5	1	10	2	11.1
Advanced course	0	0	0	0	0	0
Completed certification	1	12.5	0	0	1	5.6
Perform robotic surgery						
No	8	100	10	100	18	100
Yes	0	0	0	0	0	0

**Table 3:** Training program and models evaluation

Virtual reality exercises						
What value do Virtual exercises have for robotic surgery skills acquisition?						
1—Not important	2—Important	3—Essential				
1	6	11				
Which model is better for robotic surgery skills acquisition? Virtual exercises or anatomical models?						
1—VR is better	2—Anatomical models are better					
5	13					
Procedure models						
You consider the model used on the course as adequate for this simulation						
1—Totally disagree	2—Disagree	3—Neither agree or disagree	4—Agree	5—Totally agree	Mean	SD
0	0	0	10	8	4.44	0.511
How do you evaluate the quality of the Bariatric procedures simulation?						
1—Very bad	2—Bad	3—Neither good or bad	4—Good	5—Excellent	Mean	SD
0	0	0	5	3	4.38	0.518
How do you evaluate the quality of the Inguinal TAPP simulation?						
1—Very bad	2—Bad	3—Neither good or bad	4—Good	5—Excellent	Mean	SD
0	0	0	5	5	4.5	0.527
How do you evaluate the quality of the Ventral TAPP simulation?						
1—Very bad	2—Bad	3—Neither good or bad	4—Good	5—Excellent	Mean	SD
0	0	0	8	2	4.2	0.422

**Table 4:** General evaluation

Do you think your laparoscopic skills had influence in robotic skills acquisition?						
1—No	2—Yes, very little	3—Yes, little	4—Yes, some	5—Yes, a lot	Mean	SD
0	0	0	10	8	4.44	0.511
How do you evaluate the ease of adaptation to the robot functions?						
1—Very difficult to adapt	2—Difficult to adapt	3—Neither easy or difficult to adapt	4—It was easy to adapt to SOME functions	5—It was easy to adapt to MOST of the functions	Mean	SD
0	0	1	9	8	4.39	0.608
You feel you learned the basic functions of the robotic system						
1—Totally disagree	2—Disagree	3—Neither agree or disagree	4—Agree	5—Totally agree	Mean	SD
0	0	0	9	9	4.5	0.514
You feel you learned all the functions of the robotic system						
1—Totally disagree	2—Disagree	3—Neither agree or disagree	4—Agree	5—Totally agree	Mean	SD
0	0	10	6	2	3.56	0.705
This course is important for the rest of the congress						
1—Totally disagree	2—Disagree	3—Neither agree or disagree	4—Agree	5—Totally agree	Mean	SD
1	0	3	9	5	3.94	0.998
This course is important for knowledge acquisition for the rest of the congress						
1—Totally disagree	2—Disagree	3—Neither agree or disagree	4—Agree	5—Totally agree	Mean	SD
0	1	6	11	0	3.56	0.616

**Table 5:** Course and methodology evaluation

Globally, how do you evaluate the quality of the course?						
1—Very bad	2—Bad	3—Neither good or bad	4—Good	5—Excellent	Mean	SD
0	0	0	9	9	4.5	0.514
You will recommend the course to your colleagues						
1—Totally disagree	2—Disagree	3—Neither agree or disagree	4—Agree	5—Totally agree	Mean	SD
0	0	0	0	18	5	0
The course met your expectations						
1—Totally disagree	2—Disagree	3—Neither agree or disagree	4—Agree	5—Totally agree	Mean	SD
0	0	1	13	4	4.17	0.514
You felt confident with the faculty						
1—Totally disagree	2—Disagree	3—Neither agree or disagree	4—Agree	5—Totally agree	Mean	SD
0	0	0	6	12	4.67	0.485
You felt confident with the methodology						
1—Totally disagree	2—Disagree	3—Neither agree or disagree	4—Agree	5—Totally agree	Mean	SD
0	0	0	7	11	4.61	0.502
The course will have impact in your robotic skills acquisition						
1—Very low impact	2—Low impact	3—Neither high or low impact	4—High impact	5—Very high impact	Mean	SD
0	1	3	10	4	3.94	0.802
The course will have impact in your career						
1—Very low impact	2—Low impact	3—Neither high or low impact	4—High impact	5—Very high impact	Mean	SD
0	1	5	10	2	3.72	0.752
You feel ready to start your robotic surgery pathway						
1—Totally disagree	2—Disagree	3—Neither agree or disagree	4—Agree	5—Totally agree	Mean	SD
0	0	2	10	6	4.22	0.647
You feel ready to start operating with a robot						
1—Totally disagree	2—Disagree	3—Neither agree or disagree	4—Agree	5—Totally agree	Mean	SD
0	5	8	5	0	3	0.767
The course increased your interest in Robotic Surgery						
1—Totally disagree	2—Disagree	3—Neither agree or disagree	4—Agree	5—Totally agree	Mean	SD
0	0	1	3	14	4.72	0.575

### Statistical analysis

All statistical analyses were performed using SPSS (IBM SPSS Statistics for Windows, Version 28.0). Descriptive data were presented as frequencies, means and standard deviation.

## Results

### Pre-course questionnaire

A total of 21 participants (*n* = 21) participated in the courses. In total, there were 8 male (38%) and 13 (62%) females participants. Nine trainees were present at the "*IFSO-EC & Young IFSO course: robotic metabolic/bariatric surgery"*; and 12 at the "*4th European Hernia Society Robotic Discovery pre-course"*. Only 22.2% (*n* = 4) were residents and 13 participants (72.2%) had no experience in RS. More than 83% (*n* = 15) of the participants had robotic systems at their hospital but none of them performed RS. Other characteristics of the participants can be found on Table 2.

### Post-course questionnaire

The rate response to the post-course questionnaire was 85.7% (*n* = 18).

### Training program and models evaluation

#### Virtual reality exercises

More than 94% (*n* = 17) of the participants evaluated the VR exercises as very important or essential for robotic skills acquisition. Although this opinion was generalized, when they compared VR to anatomical silicone models, 72% (*n* = 13) of them considered the anatomical models better than VR for robotic skills acquisition—Table 3.

#### Silicone procedure models

All the models (Sleeve/Bypass, Inguinal TAPP and Ventral TAPP) were considered as very adequate for this type of simulation/course (*M* = 4.44, *SD* = 0.511) and quality of the simulation was considered very high (*M* = 4.38,* SD = 0.518*; *M* = 4.5, *SD = 0.527*, *M* = 4.2, *SD = 0.422*, respectively)—Table 3.

#### Methodology and courses evaluation

All of the trainees agreed that previous laparoscopic skills have an influence in robotic skills acquisition. Seventeen (94%) trainees consider that this methodology allowed for an easy adaptation to the robotic system and, at least, to learn its basic functions. The majority of the participants consider this kind of pre-congress course is important for the rest of the following congress and agree that it will have impact on their understating and knowledge acquisition during the congress sessions—Table 4.

All trainees rated the global quality of the courses as good or excellent (*M* = 4.5, *SD = 0.514*) and considered that the course exceeded their initial expectations (*M* = 4.17, *SD = 0.514*). Confidence was very high regarding the course faculty and methodology (*M* = 4.67, *SD = 0.485*; *M* = 4.61, *SD = 0.502*, respectively). After the course, trainees considered that this course will have impact on their robotic skills and their career but only 8 (44%) participants agree/totally agree they are ready to begin their robotic pathway (*M* = 3.39, *SD = 0.926*). Only five of them (27.7%) agree on being ready to operate on a real patient using a robot after this course (*M* = 3, *SD = 0.767*). Fourteen participants (78%) totally agree that participation on these courses increased their interest in Robotic Surgery (*M* = 4.71, *SD = 0.575*)—Table 5.

## Discussion

Simulation is key for better surgical outcomes and patient safety [12, 13]. As in general surgical simulation, there are some challenges that are specific of RS training [2, 14]. Availability of systems, costs for carrying systems around for congresses and logistics related to size and weight of the systems, are just some of those. Other important particularities of RS training is that the number of opportunities is lower than open surgery (OS) or laparoscopic surgery (LS) [15]. In general, simulation is expensive, but when we talk about OS or LS, we can simulate some basic, intermediate and even some complex procedures, using various types of models such as animal ex-vivo organs, animal skin or inexpensive silicones. Even for LS, we can use cheap lap boxes to practice [16]. When talking about RS training, we need a robot or, at least, VR simulators such as the DVSS® or the FlexVR® (Surgical Science AB, Sweden) to mimic the use of the system, and these are expensive. Additionally, RS training only allows one trainee at a time [10]. On the other side, a great feature of the robotic console is the virtual exercises module that can be used when the robot is not being used for surgery. It delivers a lot of exercises and modules that allow the trainee to learn and master every function of the system as well as objective assessment [11]. Even though, the robots in hospitals are not fully available for training, unless there is a dedicated robot or console for that purpose. Thus, surgeons get those opportunities mostly in congresses, and that is why the presence of robots is so important at scientific meetings. The outcomes we found in this study, although they are the result of the questionnaires and only represent participants’ personal opinions, are interesting for the evaluation of the impact of these robotic surgery training opportunities. As we could observe in the study, more than 94% (*n* = 17) of the participants evaluated the VR exercises as very important or essential for robotic skills acquisition. Despite of the interest of VR exercises, a surgeon tends to prefer a real model, with more or less fidelity, specifically if it mimics anatomical structures and landmarks to simulate a real procedure. More than 72% (*n* = 13) of our trainees considered the anatomical models are better than VR for robotic skills acquisition. Virtual reality exercises and the metrics that can be collected from  the console, can be a very good way to analyze skills acquisition, objectively. We didn’t do such analysis for three main reasons: first, these were the first 2 editions of the courses and they served as “pilot courses” to test the concept. Secondly, the models used, although they were very proximate, they were not the definitive versions yet, as this was their first real-life test. Finally, for assessing objective outcomes (such as console metrics, second attempt procedures, …) we would need longer courses and with less participants to have more console time for each one. This is something we can do outside of courses, otherwise it could be frustrating for the trainees.

Previous methodologies used dry-lab and unspecific models for this kind of pre-congress courses and exhibition booth demonstrations. Animal or cadaver models are not adequate to be used at a meeting venue [17]. Silicone and other dry-lab models have a good acceptance among residents and surgeons [10, 18] and they may be the most adequate models for these kind of training opportunities. Depending on the complexity of the model they can be inexpensive and easy to produce, use and discharge. Of course the more complex the model is, the higher the cost [19] but it will always be cheaper and more cost-effective than a cadaver for this kind of low/middle-fidelity simulation. Having this in mind, we wanted to update the methodology of the established courses by adding silicone procedure-specific models to the hands-on sessions. We aimed to add more fidelity to the simulation, the ability to get familiar with the robotic cart and the ports’ placement, without a significant increase on costs. For these three sessions, we kept the same distribution of the theoretical and hands-on sessions of previous courses. Three different silicone models were used, aiming to replicate the anatomy and the procedures’ steps as best as possible, to assure a good quality simulation. Despite of being preliminary versions, they were complete models and we think they accomplished the learning objectives. The results showed that the participants consider these type of models of very high quality and adequate for this kind of simulation. Data collected about the models and their assessment scale will be of extreme importance to improve the models to their final versions. Participants also considered that the methodology was very adequate and that the course will have a positive impact on the following congress, with an increase on their learning and understanding of the congress sessions. Course quality was considered good/excellent for 100% of the participants and in general, they were very confident about the faculty and the methodology. As expected for a *Robotic Surgery Discovery Course*, most of the participants had no robotic experience. Interestingly, most of them considered that this type of course had increased their interest for RS and will have an impact on their robotic skills acquisition and their career. It is important to mention that, from an educational point of view, the majority considered the course had increased their confidence for starting their RS pathway and they felt more confident to perform their first robotic procedure. On the other side, as expected for an introduction course and for inexperienced surgeons in RS, only a few felt ready to operate a patient using the robot after the course. This fact shows that RS certification and continuous practicing, including other type of simulation models, are essential to start using the robot in surgical practice.

### Study limitations

We consider that the main limitation of this study concerns the relatively small size of our sample. This is due to the reasons we pointed out before, regarding the challenges faced by RS training. This type of course cannot include many trainees per activity, because the robot only allows for one trainee at-a-time. Fortunately, there were two full robotic systems and two extra consoles per session, otherwise the group would have to be even smaller or the course had to duplicate its duration. In the present case, we think our sample is adequate, in the context of our activity, as we had to adapt to the pre-Congress characteristics—space, costs and limited time. In fact, even that the sample may look small, we were able to test the methodology and three different models on three sessions, on two different congresses (IFSO and EHS 2024 congresses). We could have divided the group in two and repeat the exercises to have more cases, but we did not want to do it because repetition has influence on the training outcomes. We wanted inexperienced trainees to test the models and methodologies, for one time only and then have their opinion.

### Study strengths

The fact that our models are activity-specific plays an important role in trainees' adherence to the course. The features and characteristics of the models, mainly the presence of anatomical structures and landmarks, allowed the trainees to use the majority of the robot functions, including staplers and ligation devices, especially in regard to the bariatric model. The ventral and inguinal models allowed them to practice peritoneum dissection, defect suture and mesh placement and we think this is a great upgrade on the course methodology, adding fidelity to the courses. This study will help us to improve the silicone models and it will be useful as a  basis for the definitive courses where we plan to use the console metrics as objective assessment tools, among others. We hypothesize that these kind of courses and models can shorten the learning curve so all of this knowledge will allow us to do some further research on the courses’ educational impact, which was not the outcome of the present study. Finally, we could also test the assessment tool and we collected some data to improve its concept. We believe it will be a major improvement in skills assessment, in the future.

## Conclusions

*Robotic surgery discovery courses* have demonstrated interesting results through the use of this innovative methodology. A training program composed by theoretical sessions, regarding the fundamentals of RS and the basics of the robotic system, and hands-on sessions has been very well accepted by the trainees. The use of silicone procedure-specific models has shown to have an important impact on the adherence and confidence on the course and methodology. The framework has proved to be an invaluable hands-on experience, allowing participants to practice and refine their skills in a realistic and controlled environment. In our opinion, these results are very satisfactory and open a door to many other studies in regard to the development of new RS training methodologies to increase access to training. These kind of activities result in increased number of training opportunities at the same time they increase confidence and interest in robotic surgery.

## Data Availability

All data access is granted upon request to the authors.

## References

[1] Puliatti S, Amato M, Mazzone E, et al. Development and Validation of the Metric-Based Assessment of a Robotic Dissection Task on an Avian Model. Journal of Surgical Research 2022;277:224-34. 10.1016/j.jss.2022.02.056 35504150 10.1016/j.jss.2022.02.056

[2] Chatterjee S, Das S, Ganguly K, et al. Advancements in robotic surgery: innovations, challenges and future prospects. Journal of Robotic Surgery 2024;18(1):28. 10.1007/s11701-023-01801-w 38231455 10.1007/s11701-023-01801-w

[3] Cepolina F, Razzoli R. Review of robotic surgery platforms and end effectors. Journal of Robotic Surgery 2024;18(1):74. 10.1007/s11701-023-01781-x 38349595 10.1007/s11701-023-01781-x PMC10864559

[4] Gamal A, Moschovas MC, Jaber AR, et al. Clinical applications of robotic surgery platforms: a comprehensive review. Journal of Robotic Surgery 2024;18(1):29. 10.1007/s11701-023-01815-4 38231279 10.1007/s11701-023-01815-4

[5] Sheetz KH, Claflin J, Dimick JB. Trends in the Adoption of Robotic Surgery for Common Surgical Procedures. JAMA Netw Open 2020;3(1):e1918911. 10.1001/jamanetworkopen.2019.18911 31922557 10.1001/jamanetworkopen.2019.18911 PMC6991252

[6] Fisher RA, Dasgupta P, Mottrie A, et al. An over-view of robot assisted surgery curricula and the status of their validation. International Journal of Surgery 2015;13:115-23. 10.1016/j.ijsu.2014.11.033 25486264 10.1016/j.ijsu.2014.11.033

[7] Azadi S, Green IC, Arnold A, et al. Robotic Surgery: The Impact of Simulation and Other Innovative Platforms on Performance and Training. J Minim Invasive Gynecol 2021;28(3):490-95. 10.1016/j.jmig.2020.12.001 33310145 10.1016/j.jmig.2020.12.001

[8] Vierstraete M, Simons M, Borch K, de Beaux A, East B, Reinpold W, Stabilini C, Muysoms F (2022) Description of the current Da Vinci® training pathway for robotic abdominal wall surgery by the European Hernia Society. J Abdom Wall Surg. 10.3389/jaws.2022.10914 38314150 10.3389/jaws.2022.10914 PMC10831684

[9] Kassite I, Bejan-Angoulvant T, Lardy H, et al. A systematic review of the learning curve in robotic surgery: range and heterogeneity. Surg Endosc 2019;33(2):353-65. 10.1007/s00464-018-6473-9 30267283 10.1007/s00464-018-6473-9

[10] Gonçalves MR, Morales-Conde S, Gaspar Reis S, et al. RAWS4all project: validation of a new silicone model for robotic TAPP inguinal hernia repair. Surgical Endoscopy 2024;38(3):1329-41. 10.1007/s00464-023-10592-y 38110794 10.1007/s00464-023-10592-y PMC10881695

[11] Gonçalves MR, Novo de Matos J, Oliveira A, et al. Robotic4all project: Results of a hands-on robotic surgery training program. Laparoscopic, Endoscopic and Robotic Surgery 2023;6(1):1-8. 10.1016/j.lers.2023.01.002

[12] Enani G, Watanabe Y, McKendy KM, et al. What are the Training Gaps for Acquiring Laparoscopic Suturing Skills? J Surg Educ 2017;74(4):656-62. 10.1016/j.jsurg.2016.12.004 28385488 10.1016/j.jsurg.2016.12.004

[13] Thinggaard E, Konge L, Bjerrum F, et al. Take-home training in a simulation-based laparoscopy course. Surg Endosc 2017;31(4):1738-45. 10.1007/s00464-016-5166-5 27515838 10.1007/s00464-016-5166-5

[14] Raison N, Poulsen J, Abe T, et al. An evaluation of live porcine simulation training for robotic surgery. J Robot Surg 2021;15(3):429-34. 10.1007/s11701-020-01113-3 32654091 10.1007/s11701-020-01113-3 PMC8134281

[15] Gonçalves MR. Robots are coming! Welcome the robots! Journal of Robotic Surgery 2024;18(1):310. 10.1007/s11701-024-02071-w 39105984 10.1007/s11701-024-02071-w

[16] Moreira-Pinto J, Silva JG, Ribeiro de Castro JL, et al. Five Really Easy Steps to Build a Homemade Low-Cost Simulator. Surgical Innovation 2013;20(1):95-99. 10.1177/1553350612440508 22434377 10.1177/1553350612440508

[17] Stefanidis D, Yonce TC, Green JM, et al. Cadavers versus pigs: which are better for procedural training of surgery residents outside the OR? Surgery 2013;154(1):34-7. 10.1016/j.surg.2013.05.001 23809483 10.1016/j.surg.2013.05.001

[18] de Montbrun SL, Macrae H. Simulation in surgical education. Clin Colon Rectal Surg 2012;25(3):156-65. 10.1055/s-0032-1322553 23997671 10.1055/s-0032-1322553 PMC3577578

[19] Fairhurst K, Strickland A, Maddern GJ. Simulation Speak. J Surg Educ 68:382–386. 10.1016/j.jsurg.2011.03.003 21821217 10.1016/j.jsurg.2011.03.003

